# A Narrative Review of Empirical Literature of Behavioral Activation Treatment for Depression

**DOI:** 10.3389/fpsyt.2022.845138

**Published:** 2022-04-25

**Authors:** Xiaoxia Wang, Zhengzhi Feng

**Affiliations:** ^1^Department of Basic Psychology, School of Psychology, Army Medical University, Chongqing, China; ^2^School of Psychology, Army Medical University, Chongqing, China

**Keywords:** behavioral activation treatment, depression, guided self-help intervention, treatment manual, e-mental health

## Abstract

Grounded in the profound tradition of behaviorism theory and research, behavioral activation (BA) has become a standalone psychotherapy for depression. It is simple, straightforward, and easy to comprehend, with comparable efficacy to traditional CBT, and has developed into an evidence-based guided self-help intervention. The main work in the theoretical models and treatment manuals, as well as empirical evidence of the effectiveness of BA for (comorbid) depression in primary and medical care setting are introduced. With the rise of the third wave of CBT, therapeutic components across diagnoses will be incorporated into BA (e.g., mindfulness). Extensive studies are required to examine the neurobiological reward mechanism of BA for depression, and to explore the feasibility and necessity of e-mental health BA application into the public healthcare system in China.

## Introduction

Behavioral activation (BA) has emerged as an effective intervention for major depressive disorder, ever since the unique role of BA in Beck' s cognitive therapy (CT) was recognized, in which BA is found to be as effective as the whole CT package (including interventions targeting autonomic dysfunctional thoughts and core beliefs, as well as BA) (1), with the latter subsequently evolving into cognitive behavioral therapy (CBT). In the subsequent literature, BA was described as a standalone therapy for depression (2). Behavioral activation is proposed as a common mechanism of change across different therapeutic approaches and disorders (3), which encourages the depressed patients to engage in anti-depressant activities which is reinforced and sustained. These behavior change may explain the enduring effects of BA in the prevention of relapse for depressed patients (4). For patients with moderate to severe depression, BA is more effective than CT (5). Contemporary BA also include many variations which were regarded as a “third wave” CBT, and focused on contextual factors and functional analysis of behaviors in assessment and intervention of depression (6). This review aims to present a conceptual overview of the BA followed by reviewing empirical evidences supporting its efficacy for treatment of depression.

## Psychopathological Models of Behavioral Activation Therapy

The model of behavioral activation (BA) is derived from the groundbreaking theoretical and empirical work of behaviorist in explanation and treatment of depression, typically Ferster (functional analysis of behavior) (7), Lewinsohn and Libet (8) (pleasant events scheduling), Rehm et al. (9) (self-control model of depression) and Beck et al. (10) (cognitive therapy and its BA component). These models can be classified as response- and action-based models. (1) The response model offers a mechanistic explanation for the causes of behavior, which regards behavior as a type of response, including physiological reflexes, ecological fixed behavior patterns, and conditional and non-conditional psychological responses. (2) The action model offers a teleological explanation that behaviors are not triggered by stimuli but are controlled by animals' perceptions of the consequences of actions. Therefore, changes in motivation or a new understanding of goals and values can lead to changes in goals and values (11).

### Response-Based Reinforcement Model

According to the response-based reinforcement model, the loss, reduction, or long-term low level of positive reinforcement leads to behavioral and emotional changes in depressed individuals. Lewinsohn believes that the withdrawal/reduction in response-contingent positive reinforcement (RCPR) can fully explain the occurrence of depression, and pleasant activity schedule could be recorded and function as positive reinforcements (8, 12). Rehm et al. (9) proposed a low self-control model of depression, which is characteristic of low self-reinforcement and high self-punishment levels. The absence or the withdrawal of (external or internal) reinforcement leads to the extinction of behaviors. Generalization of such extinction to other contexts will produce behaviors similar to absence of anhedonia; this behavior model is known as extinction-induced depression (EID) (13). Low positive reinforcement model was supported by empirical evidence that the increase in RCPR leads to decrease in depressive symptoms with BA as the mediator (14).

In contrast, other researchers have focused on the role of negative reinforcement on avoidance and withdrawal. Ferster et al. (7) argues that the withdrawal of discriminative stimuli can lead to avoidance and withdrawal, and that depression is caused by negative reinforcement of avoidance and escape behavior (15). Therefore, the aim of behavioral activation therapy is to break the trigger-response-avoidance pattern (TRAP) and promote the trigger-response-alternative cope (TRAC) pattern (16), which helps individuals adopt an alternative adaptive pattern to cope with adverse stimuli.

To sum up, while both avoidant behavior and less activating behavior contribute to decreased social contact which then reduce positive reinforcements (17), activity scheduling and monitoring of activity strategies in contemporary BA approach targets both behaviors.

### Action-Based Model

As the cognitive revolution took hold in 1970-1980's, the role of cognitive components such as goals and values in activating behaviors was emphasized according to the action-based model. BA was incorporated into Beck et al. (10) CT and formalized into daily activity (not just pleasant) scheduling, assessment and monitoring. Particularly, activity scheduling as a common treatment component during BA showed large effect size in a meta-analysis of randomized effect studies (including 60 studies with 780 subjects) when compared between intervention and control condition (Cohen' s d = 0.87) (18). Apart from the actual rewards received (positive reinforcements), the expectation of possible rewards may play an important role in reinforcement of behavior. If the reward expectation is low, individuals will demonstrate sloth, i.e., lower tendency for individual action, which then diminishes the individual's ability to receive rewards and sustains his/her incorrect estimate of the average environmental rewards. Therefore, a low expectation of rewards can explain how depressive behaviors are sustained (19).

Action-based model was supported by the clinical trial study which found that expected pleasure is the most significant factor in predicting the decrease in depressive symptoms after BA, with greater predictive power than the actual rewards received (20). The neuroimaging studies also supported the involvement of both reward expectation and consumption brain regions during BA, including the caudate during the stage of expected rewards, the putamen during the stage of action selection, and the medial orbitofrontal cortex (mOFC) and dorsolateral prefrontal cortex (DLPFC) during the stage of reward feedback (21, 22). Until recently, the neuroscience of reward processing and its formalization with computational models such as reinforcement learning (RL) greatly inspired the BA psychological model and informed the mechanism of change of BA (23, 24).

## Manualized Behavioral Activation for Depression

The manualized behavioral activation for depression was developed based on response- and action-based model to achieve the following primary goals: (1) discern the living environment that induces depression; (2) clarify and reduce the maladaptive coping patterns that sustain and exacerbate depression; and (3) improve the adaptive coping patterns which increase exposure positive reinforcement in the environments bringing pleasure or the sense of control (25).

As the earliest study dismantling BA component from CT as the standalone treatment for depression, Jacobson's regimen includes 20 sessions that involve: (1) monitoring daily activities; (2) assessing the pleasure and sense of control associated with various activities; (3) assigning increasingly difficult tasks that may reduce pleasure or the sense of control; (4) conducting cognitive rehearsals, during which patients imagine themselves participating in various activities to identify factors that may prevent patients from gaining pleasure or the sense of control from the activities; (5) discussing specific issues such as sleeping difficulty, and behavioral techniques to deal with these issues; and (6) taking measures to improve social skills such as self-confidence and communication skills (1). On the basis of Jacobson's regimen, Martell et al. (2) developed a more comprehensive manual and included other intervention strategies for behavioral activation, such as repeated freedom from issues or unpleasant events (distraction technique), mindfulness training, and self-reinforcement (2). This manual originally developed for adult depression was modified into the Adolescent Behavioral Activation Program (A-BAP) to treating depressed adolescents (age 12–18) which typically consists of 12–14 sessions (26).

Alternatively, Lejuez et al. (27) developed a brief, 12-session behavioral activation treatment for depression (BATD) that includes daily monitoring, social support contracting, activity selection and scheduling, and post-activity rewards. In contrast to BA manuals of Jacobson and Martell et al. which aim to explicitly increase approach behavior as an alternative to depressant (e.g., avoidant/ruminative) behaviors, BATD predicted that increase in reinforcement for healthy (e.g., approach) behaviors could lead to decrease in depressant behaviors and thus increase healthy behaviors, based on the matching law. BATD is more suitable for depressed patients indicated for brief and well-defined treatment (28).

A decade later, Lejuez published the revised BATD treatment manual (BATD-R) (29), which shortens the number of sessions from 12 to 10. Detailed revisions include: (1) more emphasis on the basic principles of treatment, including client-therapist relations; (2) clearer descriptions of life, values, and activities; (3) simplified and fewer treatment sessions; (4) more details about the program, including a concept review of obstacle elimination and late-stage treatment; and (5) a new revised daily monitoring schedule for patients with low education levels (30). Lejuez et al. state that the number of sessions may be adjusted according to the patient and clinical context.

Single session BA treatment was also developed for college students who significantly reduced depressive symptoms after they received the single BA session (31). During a 90-min session, the following issues will be covered: psychoeducation about depression which depend on behavioral activation model, and life areas consistent with individualized values/goals, activity scheduling according to life areas which will be monitored to be completed within 2 weeks after the session. Computerized single-session BA was developed for high school students, which was appraised as acceptable and feasible by students and educators (32).

## Empirical Evidence of Behavioral Activation for Depression

### Search Strategy

We searched Willey Online Library, Sciencedirect, APA PsycArticles, Karger and BMJ online databases for literature of all publication types, with following search strategies: keywords for “behavioral activation & computerized,” “behavioral activation & computer,” “behavioral activation & web,” and “behavioral activation & depression.” Reference lists included articles and reviews dealing with individual-based BA for depression, and excluded CBT with no reference to behavioral activation component or BA adapted specifically for other settings such as anxiety, PTSD, chronic pain and so on. The time periods for the literature search were not specified and set as default. We manually screened the literature lists and specifically focused on clinical trials which followed the manuals of Martell et al. (33) and Lejuez et al. (29).

### Effectiveness of BA Across Depressed Groups

BA is developed for depressed patients, however, it is also efficacious within diverse depressed populations including those comorbid with other mental or physical disorders.

#### Depression

The 10-week session BA increased behavioral engagement and decreased depressive symptoms (self-reported and clinician-rated) for patients with major depressive disorder or dysthymic disorder in multi-site community with medium to large effect sizes when compared to psychosocial treatment as usual with no BA or CBT services (N_BA_ = 31 vs. N_TAU_ = 33; Cohen' s d = 0.70–1.02) (34). The BATD decreased depressive symptoms for depressed inpatients when compared to supportive psychotherapy (N_BATD_ = 10 vs. N_supportivetherapy_ = 15; Cohen' s d = 0.75) (35). A simplified version of 7-session BA translating from Lewinsohn' s pleasant events scheduling into a healthcare intervention improved mild or subthreshold depressive symptoms in elderly inpatients, with moderate effect size but not statistically significant (N_BA_ = 30 vs. N_control_ = 25; Hedges' s g = 0.35) (36, 37). Outpatients of new recruits (*n* = 21) in primary care showed improvements in anxiety and depression with large effect sizes (Cohen' s d = 1.94, g = 1.75; Cohen' s d = 1.86, g = 1.68) (38).

#### Atypical Depression

BA is also suitable for atypical depression with mood reactivity (i.e., better response to favorable events), psychomotor retardation, and interpersonal withdrawal as core symptoms. BA comprising of 14–16 sessions alleviated depressive symptoms and functional impairment for atypical depression with large effect sizes (*N* = 9; Cohen' s d = 2.8 and 2.2) (39). BA also ameliorated social withdrawal and occupational dysfunctions after medical treatment for patients with chronic depression, by activating the patient into work-related goals, with large effect-size improvement in negative symptoms (*N* = 16; d = 0.76) (40, 41).

#### Depression Comorbid With PTSD

BA is suitable for depressed patients comorbid with other mental (e.g., PTSD) or physical disorders (e.g., diabetes, cancer). BA based on the manual of Martell et al. (2) reduced depression and posttraumatic stress disorder (PTSD) scores with moderate effect sizes (*N* = 20; Cohen' s d = 0.36 and 0.47) (42). BA also alleviated pathological grief, PTSD, and depressive symptoms in middle-aged individuals undergoing bereavement (N_BA_ = 13 vs. N_waitlist_ = 12; Cohen' s d = −1.19) (43), and elderly individuals undergoing complicated bereavement significantly improved on PTSD and depressive symptoms (*N* = 26; partialη^2^ = 0.41 and 0.38) (44). BA has also been effectively used in occupational populations with depressive disorder, especially those comorbid with PTSD. Behavioral activation and therapeutic exposure (BA-TE) demonstrated improvements in symptoms of PTSD (*N* = 117, β = −0.72, *P* < 0.01), and overlapping symptoms of PTSD and depression (*N* = 117, β = 0.81, *P* < 0.01) for patients with depression associated with PTSD (45). BATD via electronic communications for active-duty US military service members and older adult veterans with depression showed both improvements in quality-adjusted life-years (QALYs) and cost benefits (46, 47). A 12-week group BA treatment yielded recovery or improvement (according to Jacobson-Truax Classification) in depressive (58% of all participants) and PTSD symptoms (65% of all participants) in veterans (48).

#### Depression Comorbid With Diabetes

For patients with type 2 diabetes and depression (n_BA_ = 15 vs. n_control_ = 14), a combination of BATD and fitness routine improved depressive symptoms (Beck Depression Inventory-II, Cohen' s d = −0.49; Hamilton Depression Rating Scale, Cohen' s d = −0.66) over time with moderate levels of evidence (49, 50). The increase of the behavioral activation system (BAS) was significantly stronger after BA than Supportive Psychotherapy (SP) intervention (N_BA_ = 36 vs. N_SP_ = 33; Cohen' s d = 0.61) for elderly depressed patients with diabetes, with BAS as a predictor of the decrease of BDI scores (51). These results supported BA' s effects in facilitating active coping and increasing approach behaviors in depressed patients suffering from chronic physical disabilities, which consequently improved their life quality and medical outcome.

#### Depression Comorbid With Cancer

BA encouraged the cancer patients to engage in active coping, decreased depression and suicidal ideation, and increased hopefulness and life quality after BA treatments (52, 53). A 9-session BATD for cancer outpatients comorbid with depression in primary care setting improved symptoms of depression and anxiety, quality of life, and treatment outcomes with moderate to large effect sizes (*N* = 6; Cohen' s d = 1.7 to 2.3) (54). A similar 8-session BATD for breast cancer patients decreased depression and anxiety symptoms (N_BA_ = 42; Cohen' s d = 1.55 to 1.91), as well as increased environmental reward and medical outcomes with moderate to large effect sizes (N_BA_ = 42; Cohen' s d = 0.33 to 0.93) (55). Longitudinal data for 8-session BATD indicated decreased depressive symptoms as depressed cancer patients completed higher proportion of scheduled activities from pre- to post-treatment with strong effect sizes (*N* = 23; high pseudo *R*^2^ values with Cox & Snell = 0.44, Nagelkerke = 0.64) (56).

To sum up, there is increasing evidence of the effectiveness of BA for both sub-threshold and clinically diagnosed depression. The effectiveness of BA in depressed patients comorbid with mental illness or physical diseases calls for more attention to strictly designed empirical studies including clinical trials with ample sample size in both primary and medical care setting.

### Web- and Smartphone-Based Interventions

Because of BA's advantage as an evidence-based low-intensity intervention, it could be easily distributed and delivered in computerized and internet-based format. The advantages of computerized and internet-based BA include privacy protection, easier access to mental services, lower travel/time costs, no waiting, and no loss of work time (57). Surveys among depressed patients suggested that depressed patients prefer guided self-help interventions and psychotherapy over medical treatment (58). The development of smart-mobile phone technology has made it possible to provide mental health services and collect data more efficiently at any time and from anywhere, which have laid the foundation for individualized mental health services (59). With the rapid development of mobile networks and more web-based mental health resources, mobile psychotherapy will become a new e-mental health model after web-based psychotherapy.

There are mainly two types of online BA program. (1) Web-based BA or integrative psychotherapy online program that includes BA component. For example, “BAML” for moderate to severely depressed individuals (25), internet behavioral activation (iBA) for postpartum depression (60), Deprexis (59), CATCH-IT (61) and its Chinese version “Grasp the Opportunity” program for adolescents in Hong Kong (62), MoodGYM (63) and its Chinese version (64). There are versions of web-based BA for subthrehold depression, for example, Beat the Blues (BTB) for poor and at-risk populations (PHQ ≥ 5) (65). (2) Smartphone-based BA program. For example, an 8-week BATD for active-duty US military service members (47), a smart phone-based BA software that includes modules on mental education, behavior selection and scheduling, behavior monitoring, and feedback (66), Mobilyze, a smartphone-based software with hardware support developed by the Pew Research Center that is the first treatment software in the world for depression with real-time ecological intervention using situation awareness to monitor mental health (67). It can predict the patient's mood, emotions, cognitive/motivational state, behavioral activities and social context on the basis of real-time ecological data collected by mobile phone sensors and classified with machine learning algorithms.

Web-based therapy makes it easier for individuals to seek psychotherapy. Due to its low-intensity treatment and easier access, it can be used as a prevention intervention to reduce depressive symptoms and negative thinking in individuals with non-clinical depression (68).

For web-based treatment, the main limitation is that it cannot be integrated into a patient's life. In addition, web-based treatment often requires a large amount of text and significant reading. Although patients can carry paper materials and contact therapists via the internet, the actual operation is not convenient.

In contrast, mobile phones are easy to carry and can monitor, record, and intervene when behaviors occur, which advantages had been summarized elsewhere (69). Additionally, mobile software is usually more user-friendly, and its web pages are more integrative than internet pages, which is more attractive and motivating for depressed patients who are prone to fatigue, and thereby reducing dropouts (66).

Notably, whereas BA component was included in most of web- and smartphone-based interventions for depression (19 out of 29 apps, 68%, searched online of popular app stores and search engines, January/2018), adherence to treatment guidelines needs to be emphasized in further research (70). In addition, qualitative research can be conducted to evaluate the feasibility, usability and acceptability around real world use of BA interventions and its online programs.

## Research Outlook

Future research should take into account the following considerations:

(1) Clarification of the Neurobiological Mechanism of Change Underlying BA Intervention. BA model postulates that insufficient or loss of positive reinforcement (reward) leads to and sustains depressive behaviors and emotions. Past studies have shown that the reward circuit was modulated by BA (21, 22). Future research needs to clarify the neurobiological mechanism of BA, such as the regulated neurotransmitters and electrophysiological activities of dopaminergic neurons in the reward neural network.

(2) Individualization of Treatment Needs. Activity scheduling is one of the key elements of BA therapy which showed robust effect size (18). The types of activities and how these activities are connected to the client' s values and life goals are important considerations when developing a treatment plan suitable to a patient's cultural background and specific needs. Artificial intelligence techniques such as machine learning can be used to detect treatment needs and meet individualized treatment requirements.

(3) Active Ingredients and Variants of BA. Behavioral activation has become a more comprehensive treatment approach with the rise of the third wave of CBT, which emphasize targeted value-based BA. Traditional oriental practices and fitness routines such as Yoga, Tai chi, Qigong, and mindfulness are being incorporated into BA to improve happiness experience (71). Therefore, more empirical research dealing with the active ingredients and variants of BA under the third wave of CBT needs to be conducted.

(4) Translation and Validation of BA in China. Overall, mental health literacy in China is increasing and there is still a gap with other countries, among domestic regions and groups (e.g., higher education and younger age) (72–74), mental health literacy could be improved through education and training in order to better understand depression and implement prevention and treatment measures. To date, empirical evidence for effectiveness and mechanism of BA is increasing rapidly in China (18 clinical trials published in Chinese, 2011~2022), and yet still limited compared with traditional and third wave CBT evidence. As the low-intensity and easily trained BA is gaining more attention, the development and translation of the manual and computerized/smartphone-based software BA is gaining more attention, for example, the introduction of internet cognitive-behavioral therapy (ICBT) for depression (MoodGYM) by Beijing Suicide Research and Prevention Center (BSRPC) (64), our translation of BA manual by Martell et al. (33) into Chinese (published in 2017, ISSN 978-7-5621-8052-4), and clinical trial of guided self-help BATD treatment for geriatric depression following the manual by Lejuez et al. (29, 75).

## Conclusions

Based on BA treatment manual following Martell et al. (33) and Lejuez et al. (29), there has been increasing evidence of BA' s effectiveness for both sub-threshold and clinically diagnosed depression, as well as depressed patients comorbid with mental illness or physical diseases. Limitations pertaining to the e-mental health modalities and the underlying mechanism of change of BA should be addressed in the future research.

## Author Contributions

XW and ZF conceived of the presented design of the study. XW collected literatures, reviewed relevant researches, and drafted the manuscript in consultation with ZF. All authors contributed to the article and approved the submitted version.

## Funding

This research was financially supported by the Youth Cultivation Foundation of Medical Science in Army Medical University (2016XPY08), the National Youth Cultivation Foundation of Military Medical Science (17QNP002), and the Chongqing Social Science Planning Project (2017QNSH21).

## Conflict of Interest

The authors declare that the research was conducted in the absence of any commercial or financial relationships that could be construed as a potential conflict of interest.

## Publisher's Note

All claims expressed in this article are solely those of the authors and do not necessarily represent those of their affiliated organizations, or those of the publisher, the editors and the reviewers. Any product that may be evaluated in this article, or claim that may be made by its manufacturer, is not guaranteed or endorsed by the publisher.
